# Artificial neural network analysis for classification of defected high voltage ceramic insulators

**DOI:** 10.1038/s41598-024-51860-8

**Published:** 2024-01-17

**Authors:** Ahmed S. Haiba, A. Eliwa Gad

**Affiliations:** Electrical Metrology Division, National Institute of Standards (NIS), Giza, Egypt

**Keywords:** Engineering, Electrical and electronic engineering

## Abstract

Partial discharge (PD) could lead to the formation of small arcs or sparks within the insulating material, which can cause damage and degradation to the insulator over time. In ceramic insulators, there are several factors that can cause PD including manufacturing defects, aging, and exposure to environmental conditions such as moisture and temperature extremes. As a result, detecting and monitoring PD in ceramic insulators is important for ensuring the reliability and safety of electrical systems that rely on these insulators. In this study, acoustic emission technique is introduced for PD detection and condition monitoring of defective ceramic insulators. A sequence of data processing techniques is performed on the captured signals to extract and select the most significant signatures for classification of defects in insulator strings. Artificial neural network (ANN) has been used to build an intelligent classifier for easily and accurately classification of defective insulators. The overall recognition rate of the classifier was obtained at 96.03% from discrete wavelet transform analysis and 88.65% from fast Fourier transform analysis. This obtained result indicates high accuracy and performance classification. The outcomes of ANN were verified by SVM and KNN algorithms.

## Introduction

**Figure 1:**
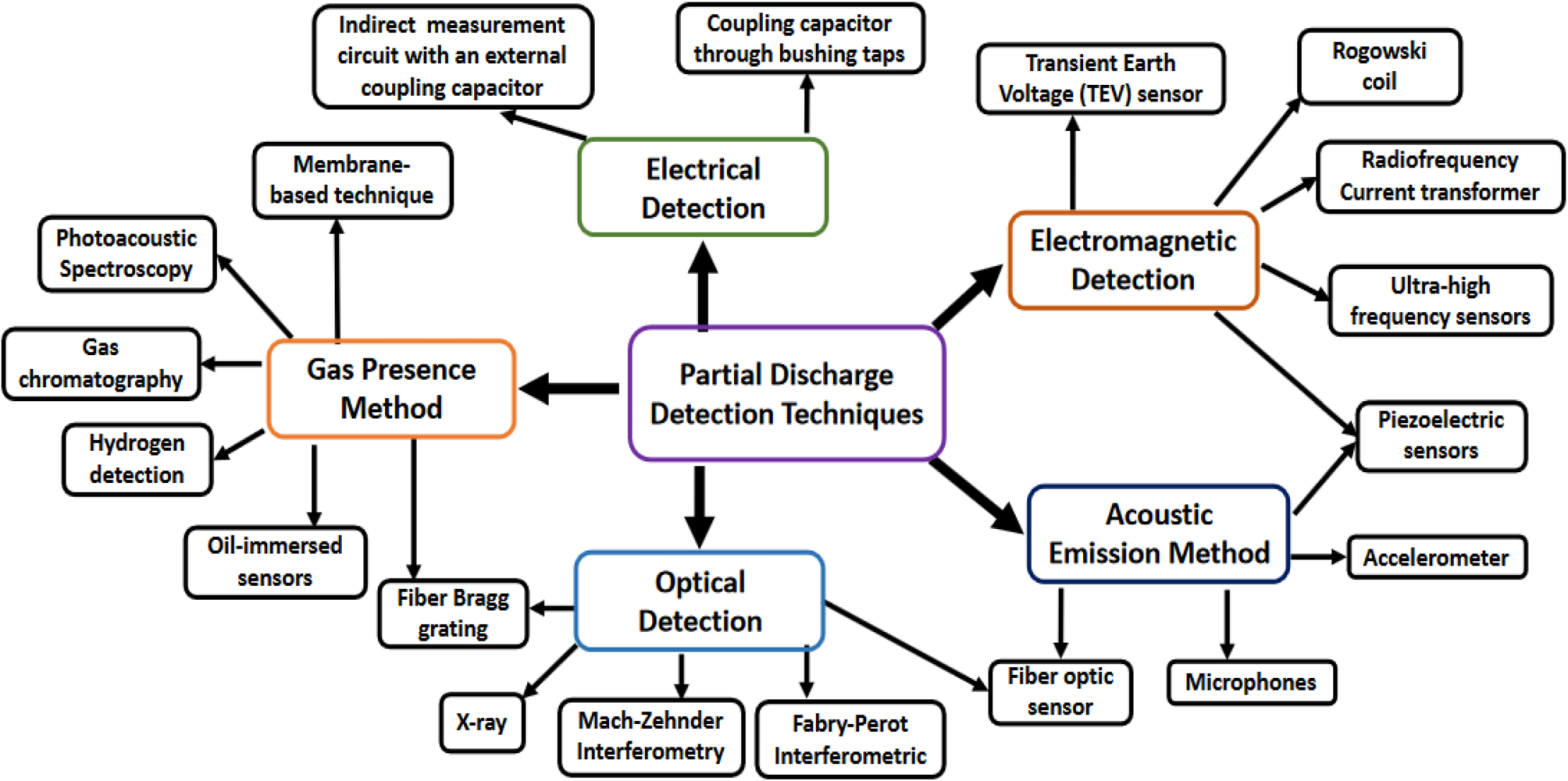
Measurement and detection techniques of partial discharge.

Utilities have used outdoor insulators to support overhead lines at both transmission and distribution voltage ratings. These insulators serve a crucial function of insulating the high voltage transmission line from the steel tower^1^. Due to their nature of operation in overhead transmission and distribution lines, outdoor ceramic insulators are subjected to various defects under operating and environmental conditions. One of these defects is the expansion of cement which can form cracks in the insulator, and this expansion can be accelerated under wet conditions leading to insulator failure^2^. As a result, Cherney and Hoton^3^ introduced water expansion tests on the used cement to ensure its quality when assembled with the insulator. Also, erosion and corrosion of pin and cap hardware in the insulator is another defect that can occur due to the salt solution formation on the insulator surface. Under wet atmospheric conditions, the formed salt solution may lead to a loss in cross-section area and mechanical strength in the insulator pin leading to the overhead conductor dropping^4^. Furthermore, contamination flashover is the main problem of overhead insulators^5–8^. Contaminants, such as salts, chemical particles, dust, sand, etc., can accumulate on the surface of the insulator. In wet conditions, the pollution surface layer is converted into a conductive layer and the leakage current passes through these layers on the insulator surface causing heating of that layer. Dry bands are then formed and discharges can continue until the insulator flashover and outage of the power line occur^9^. Ceramic insulators may be broken and punctured due to the occurrence of overvoltages from the lightning on the transmission line^4^. Also, during the manufacturing process, very small internal voids or cracks may be developed within the insulator^10,11^. These voids may cause continuous discharges under operating conditions, leading to insulator failure. As a result, PD growth measurement is the most significant indicator of all the above mentioned defects that occurred for the overhead insulators^12^. Consequently, many researchers have introduced various methods to evaluate the insulators’ condition based on PD signatures^1^. Many detection techniques, based on both electrical and non-electrical methods, have been introduced for the detection, analysis, and evaluation of PD activities^13–19^. Each technique has benefits and drawbacks, and one may be better than the other depending on the application. A brief overview of the widely used techniques for monitoring the condition of the overhead line insulators will be provided in Fig. 1^20^.

In order to create a classifier that can distinguish between and identify faults, partial discharges, defects, or degradation, a number of factors have been manually collected from recorded patterns or signals over time^21^. While the process has become somewhat automated, the need for experts to manually select the features presents a challenge, as different features may lead to different outcomes. This relying on selected features manually can adversely affect the classifier's performance.

Deep learning enables the integration of feature selection with the learning process, thereby automating the entire process. In the realm of applications of high voltage, the primary objective has been to localize or classify faults, defects, or partial discharges that could occur in high voltage equipment, or to detect the deterioration of insulating materials. Classification involves differentiating between various sources of faults, defects, partial discharges, or levels of degradation. Artificial intelligence techniques that are applied to classify the condition of insulators hold great promise, as defects could be automatically fixed out of pattern recognition^22^. Deep learning techniques can be utilized to detect the presence of a fluctuating number of missing discs in transmission lines consisting of chain insulators^23^. The main challenge in using computer vision to detect insulator failures is the infrequency of such failures. As a result, training a network to recognize specific conditions becomes challenging due to the limited dataset available^23^. However, applying engineering constraints to datasets captured through inspections could enhance the model's accuracy, resulting in an accuracy rate of up to 92.86%, as demonstrated in^24^.

Many techniques of artificial intelligence have been developed for predicting the stability of smart grids^25–29^. Furthermore, researchers have shown the widespread utilization of these techniques such as artificial neural network (ANN)^30–32^, support vector machine (SVM)^33^, fuzzy logic (FL)^34^, K-means clustering^35^, and hidden markov model (HMM)^36^ in addressing electrical power system and high voltage engineering issues^37,38^. Intelligent systems can improve the reliability of the transmission and distribution power system, reduce costs, and reduce human effort by facilitating effective assessment of the state and performance of outdoor insulators during voltage operation^39^. In their study, Salem et al.^40^ utilized insulator diameter, height, creepage distance, form factor, and equivalent salt deposit density (ESDD) as input parameters to train a model that combined the Adaptive neuro fuzzy inference system (ANFIS) with ANN. Also, by establishing correlations between leakage current and weather conditions, A. Din et al.^41^ used the SVM technique to evaluate the leakage current for outdoor insulators. In another work, Saranya et al.^42^ put forward a novel approach for assessing the status and performance of outdoor insulators, which involves recognizing the arc faults of insulators through measurements of phasor angle.

In this current work, the detection technique of acoustic emission is used for the detection of PD signatures resulting in artificial defects in ceramic outdoor insulators. A series of advanced signal processing techniques are performed on the captured signals from the experimental section to extract and select the most significant features to be as input data for the proposed classifier. Accordingly, an artificial neural network (ANN) analysis is proposed in order to assess easily, cost-effectively, and accurately the classification of defective insulators. Other algorithms such as support vector machine (SVM) and K-nearest neighbors (KNN) are used to validate the outcomes of the ANN proposed technique.

## Materials and methods

### Experimental test set up

The common defects that could occur in the ceramic outdoor insulators are the breaking and cracking of the ceramic shell dielectric material. The occurrence of these defects depends on several factors, such as the places of these insulators, contamination degree, environmental conditions, and operating stresses.

**Figure 2:**
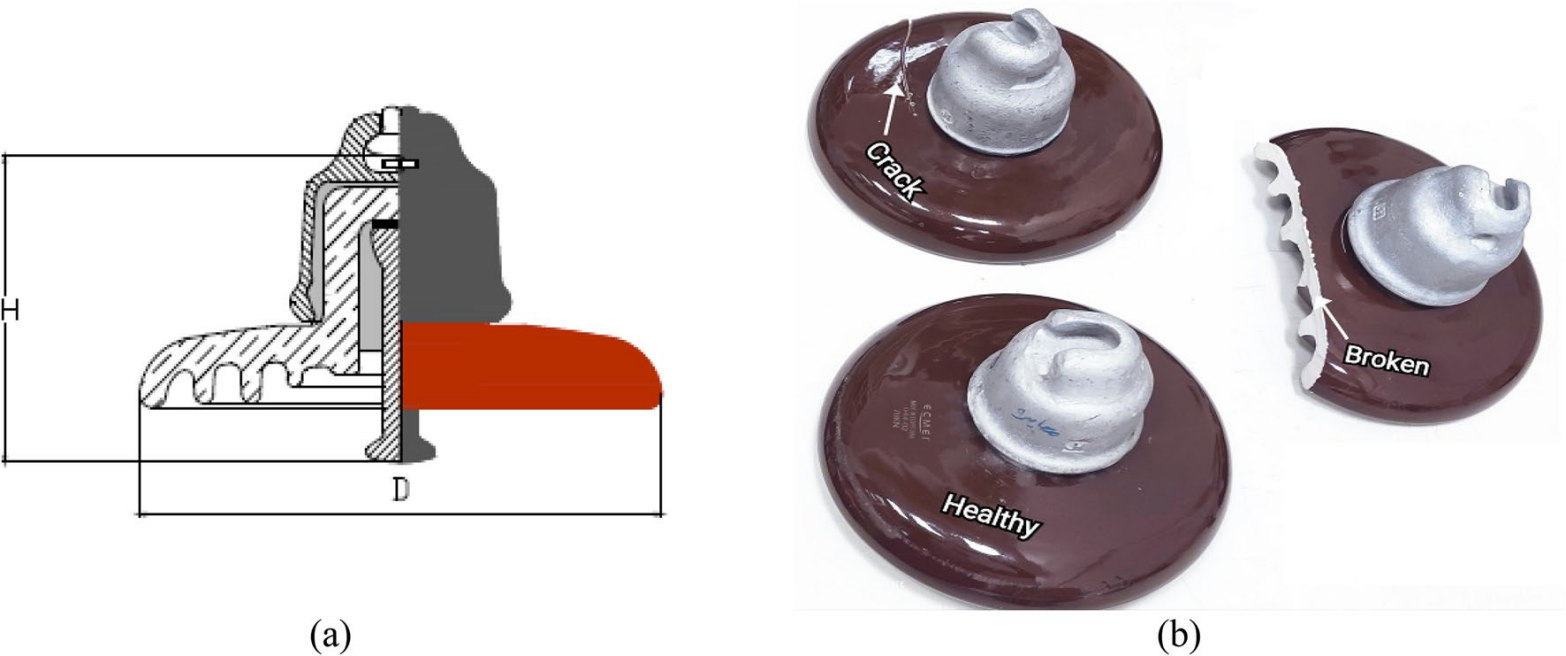
(**a**) Pin-cap insulator construction, and (**b**) Three samples of ceramic insulators: completely-broken insulator, cracked insulator, and healthy one.

Three samples of pin-cap ceramic insulators were tested in this work. Figure 2a shows the construction of the used insulator in this study that was acquired from the Egyptian Company for Manufacturing Electrical Insulators (ECMEI), Elsewedy Electric, with features^43^: 11 kV rating voltage, 255 mm diameter (D), 146 mm spacing (H), 320 mm creepage distance, and 90 kN mechanical strength. Variable artificial defects were introduced in two of them. One sample was completely broken and the other was cracked. The third one is considered the reference healthy sample without any apparent defects. Figure 2b shows the three samples tested in this study.

**Figure 3:**
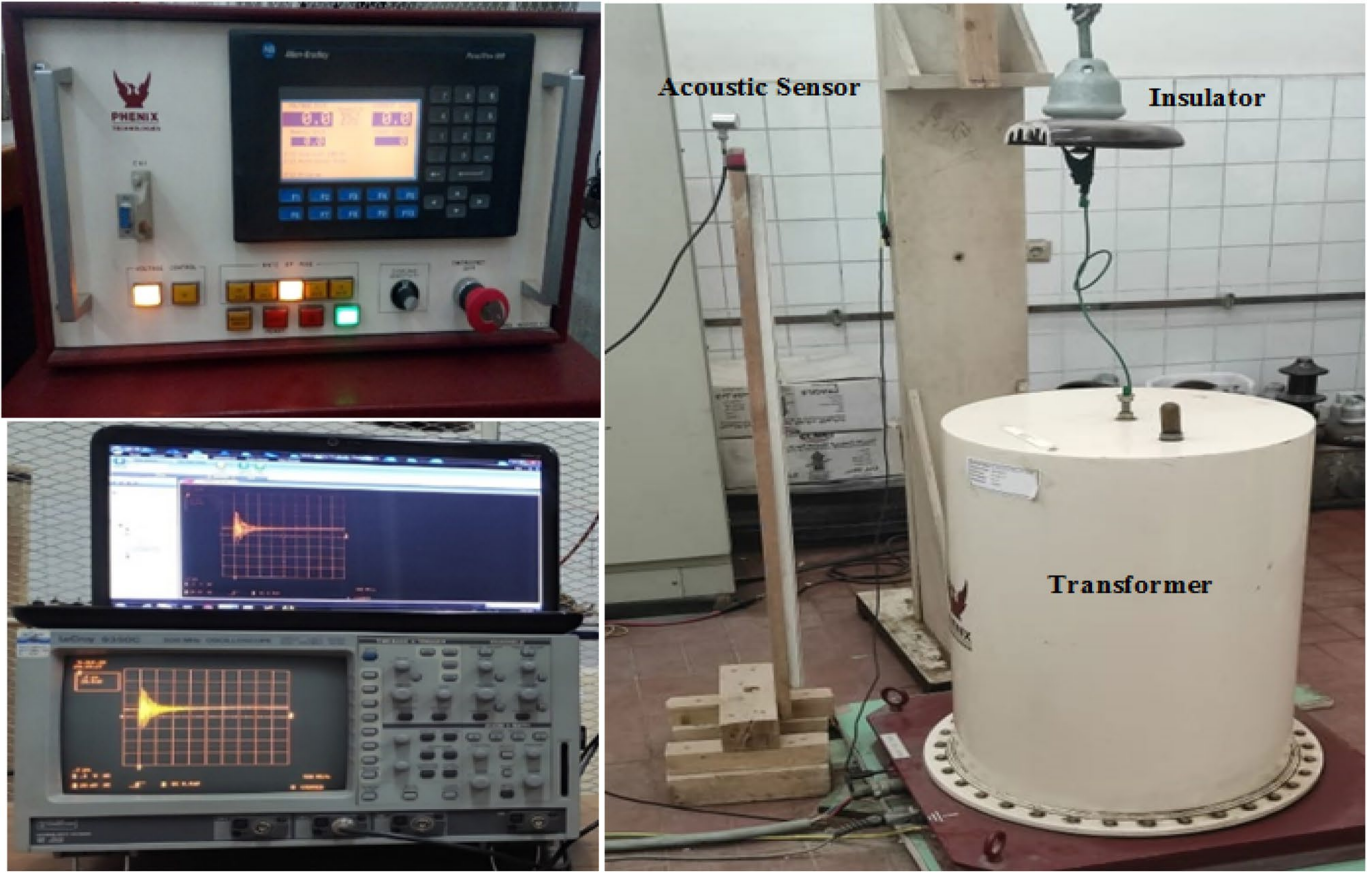
Experimental test setup.

This passage describes the experimental test setup used in this current study. The setup is shown in Fig. 3 and involves a 100 kV high voltage transformer that applies a test voltage to the samples being evaluated. The samples are tested vertically as normal operation, with the pin side connected to the high voltage terminal of the test transformer and the cap side grounded. To generate PD activities, the individual disc samples are exposed to an AC voltage of 40 kV, 50 Hz (sinusoidal waveform), in an air-insulated medium, which causes PD activities due to various defects. An 80 MHz acoustic sensor is used to capture the signals that the defective insulators emit. These signals are then transmitted via a BNC coaxial cable to a digital oscilloscope with a 500 MHz bandwidth and a 500 MS/s sampling rate for further analysis. The oscilloscope is linked to a personal computer (PC) through a GPIB cable for recording the radiated signals. 100 PD pulses are captured and recorded for each tested insulator, with each pulse comprising 4000 raw data points indicating the amplitude in mV versus time in µs.

### Feature extraction and selection

After collecting acoustic emission signals from various partial discharge (PD) activities, the next step involves analyzing these signals to establish the connection between the acquired signal and the corresponding defect. As a result, a series of procedures are applied to the collected signals in order to extract and get useful information. In this study, the acoustic-collected signals are processed in four steps: wavelet transform, feature extraction, feature selection, and classification. Furthermore, the Fourier transform technique is also used to be compared to the wavelet transform analysis tool.

Discrete wavelet transform (DWT) is used as a first stage to decompose the original signal into two groups: approximations, which reflect the signal's high-scale and include low-frequency components, and details, which indicate the signal's low-scale and include high-frequency components. DWT is mainly used as a feature extraction technique for extracting characteristics or features from the decomposed high and low-frequency signals.

Mother wavelet is selected based on the similarity between AE signal and mother wavelet based on qualitative approaches. Normally, shape matching by visual inspection is applied to pick up the most proper mother wavelet. Several researchers worked on this point of selection of mother wavelet in case of power system transients such as acoustic emissions^44–49^. W. N. A. W. Mohammad et al.^47^ concluded that the forms of wavelets db, coif, sym, bior, and rbio are used since they are the most appropriate wavelets in the case of acoustic and electrical emission of PD signals. Safavian et al.^48^ concluded that the db4, coiflet, and b-spline were equally suitable in detecting power system transients. Accordingly, these findings are supposed to be a guide for this current work.

After decomposing the original signal by DWT into 5-levels of approximation and detail signals, the features can then be extracted from each decomposed level. Each of the wavelet components that have been decomposed yields seven descriptive waveform features: mean, variance, maximum amplitude, minimum amplitude, mean of energy, skewness, and kurtosis, which are characterized as follows:

Mean (m) is defined as the average value of the number of samples (n).

1 $${\text{m}}=\frac{1}{{\text{n}}} {\sum }_{{\text{i}}=1}^{{\text{n}}}{{\text{x}}}_{{\text{i}}}$$

where: n is samples’ number and ${\text{x}}$ is the sequence of the samples.

Variance (V) is a statistical measure that quantifies the amount of variability or spread in a set of data values. It is calculated by taking the average of the squared differences of each data point from the mean.

2 $${\text{V}}=\frac{1}{{\text{n}}-1}\sum_{{\text{i}}=1}^{{\text{n}}}{({{\text{x}}}_{{\text{i}}}-{\text{m}})}^{2}$$

Maximum amplitude of a signal refers to the highest value of the amplitude that the signal reaches during its cycle.

Minimum amplitude of a signal refers to the lowest value of the amplitude that the signal reaches during its cycle.

Mean of energy (m_e_) is the energy mean value^1^ and can be calculated by taking the sum of the squares of the values in the signal, divided by the total number of values.

3 $${{\text{m}}}_{{\text{e}}}=\frac{1}{{\text{n}}} {\sum }_{{\text{i}}=1}^{{\text{n}}}{{{\text{x}}}_{{\text{i}}}}^{2}$$

Skewness (S) is a measure of the extent of asymmetry in a distribution with reference to the sample mean^1^.

4 $${\text{S}}=\frac{{\sum }_{{\text{i}}=1}^{{\text{n}}}{{({\text{x}}}_{{\text{i}}}-{\text{m}})}^{3}}{({\text{n}}-1){\upsigma }^{3}}$$

where: $\upsigma$ is the standard deviation.

Kurtosis (K) is a measure of how peaked or flat a distribution is compared to a normal distribution^1^.

5 $${\text{K}}=\frac{{\sum }_{{\text{i}}=1}^{{\text{n}}}{{({\text{x}}}_{{\text{i}}}-{\text{m}})}^{4}}{({\text{n}}-1){\upsigma }^{4}}-3$$

Following the extraction of the above seven features, the ANOVA test (the analysis of variance) was utilized as a feature selection technique to identify significant features at each level for the purpose of detecting different types of defects. Specifically, a one-way ANOVA test was employed to measure the statistical significance of differences between the means of three distinct groups: a healthy sample and two different types of defects. The variance ratio of mean squares between and within groups is the ANOVA F-distribution test statistic. The decision rule for this test is based on P-value. If the estimated P-value is more than a value of 0.05, at α-level = 0.05 with a 95% confidence level, significant differences exist between the tested groups.

### Artificial neural network

**Figure 4:**
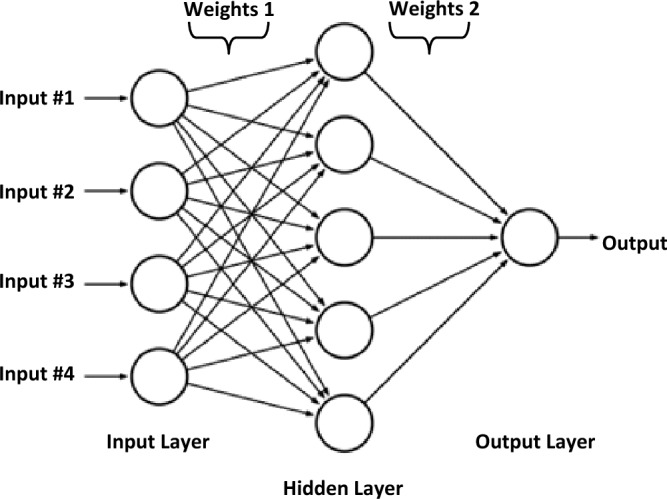
Architecture of the ANN.

The last stage of data processing is the pattern recognition or classification process, which identifies the types of PD activities. The present study utilizes an artificial neural network (ANN), commonly referred to as a neural network (NN), as an advanced classification tool for identifying various types of defective insulators. Generally, pattern recognition or classification algorithms include two stages: training, which is known as learning, and testing which is known as classification^50^. The role in the training stage is to model a mathematical relationship between the data sets and their respective PD pulses^50^. While in the testing phase, new input data points that were not part of the training data are defined as a single category of PD sources^50^. ANNs are a type of computational model that takes inspiration from the way biological neural networks in the human brain process input data. Figure 4 displays the structure of an ANN which comprises of three layers: an input layer, one or more hidden layers, and an output layer. In ANNs, a sequence of interconnected units or nodes are used, which are commonly referred to as artificial neurons. Each interlink, like the neurons in a living brain, has the capacity to transmit data or signals to other connections. These connections and neurons typically have a weight that varies as learning progresses. The weight can increase or decrease the signal intensity at a connection link. To get the neuron output, first the weighted sum of all points is taken, then a bias term is added to this sum^50^. Such a weighted sum, which is usually called the activation, is then passed via an activation function that generates the output results^50^.

### Ethical approval

This article does not contain any studies with human participants or animals performed by any of the authors.

## Results and discussion

### Acoustic emission signals

In a laboratory environment, three samples were tested at 40 kV with a sinusoidal waveform at 50 Hz. Two of the samples were defective insulators, while the other one was healthy. Using a 500 MHz oscilloscope, the shape of the discharge pulse for each test sample was captured based on the amplitude of the discharge pulse in mV vs. time or samples. This is referred to as the time-domain characteristics of acoustic signals obtained from PD activities. Using the established data acquisition system (DAS), 100 discharge sound pulses were randomly obtained and recorded for each test sample.

The recorded acoustic emission signals are subjected to a Fast Fourier Transform (FFT) tool, which converts them into their frequency domain. This process enables the frequency bands for each test sample to be shown.

**Figure 5:**
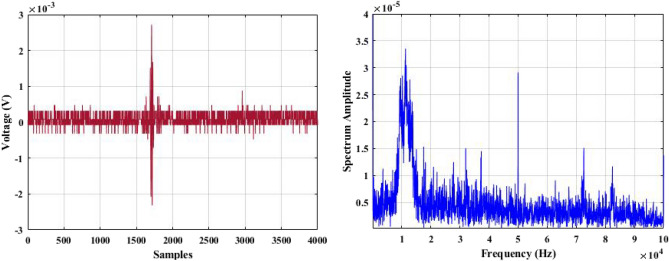
Single PD acoustic signal for a healthy insulator in time and frequency domains.

**Figure 6:**
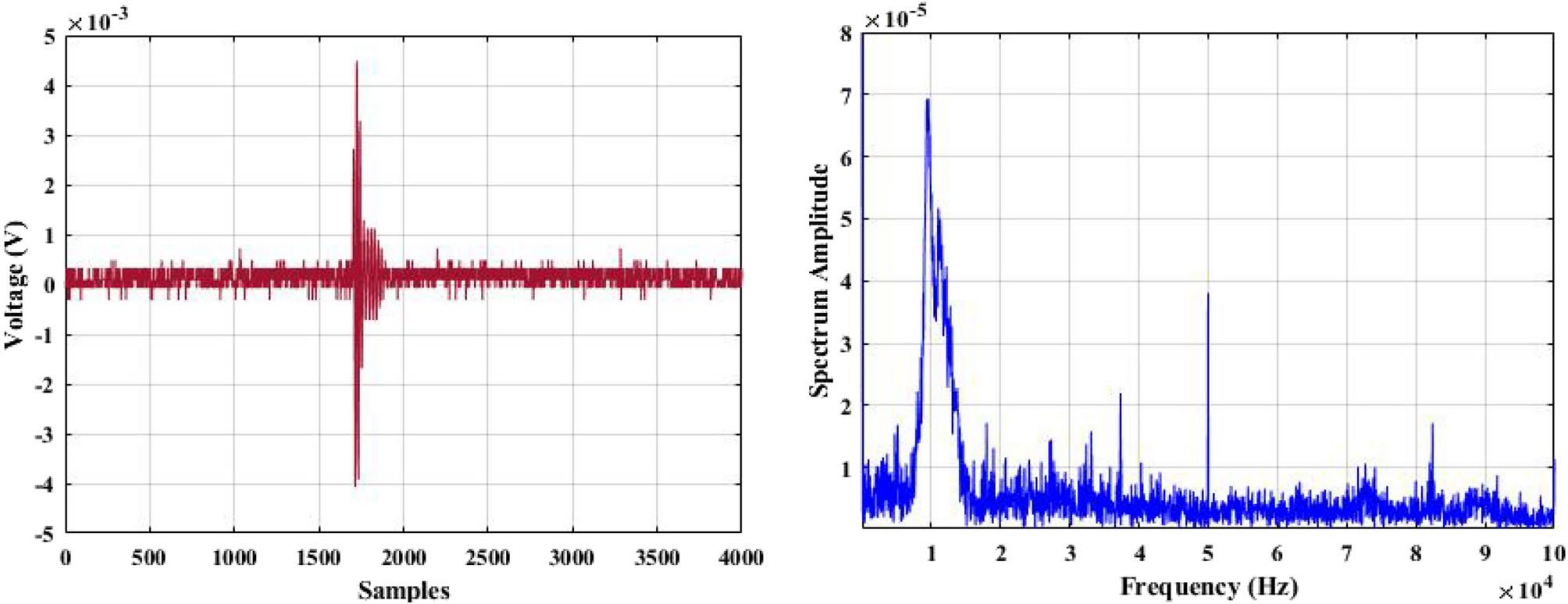
Single PD acoustic signal for a completely broken insulator disc in time and frequency domains.

**Figure 7:**
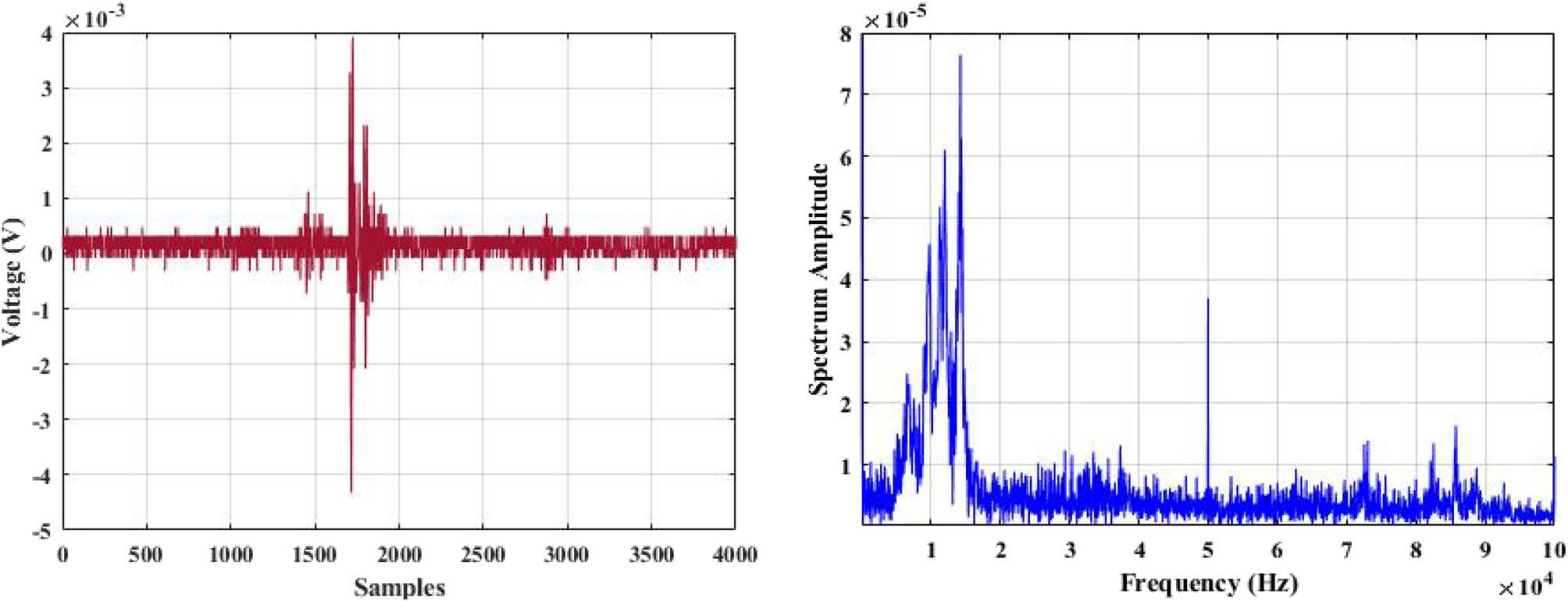
Single PD acoustic signal for a cracked insulator disc in time and frequency domains.

Figures 5, 6 and 7 display one of the collected waves for each of the three test samples in both domains: time and frequency. Visual inspection of the figures reveals differences in waveform shapes between the three test samples. Additionally, the magnitude of the PD sound pulse of the defective samples is higher than that of the healthy sample.

**Figure 8:**
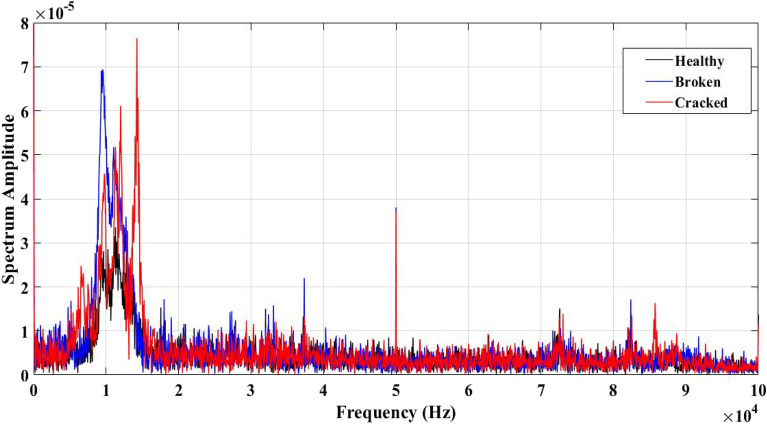
Single PD acoustic signals for all tested samples in frequency domain.

Figure 8 shows PD signatures of all samples in the frequency domain. It has been observed from this figure that peaks of the spectrum amplitude have occurred at variable frequencies for each sample. That means there are significant differences between the tested samples.

### Feature extraction and selection

Following recording, the PD acoustic emission signals gathered by the established DAS were transferred into the MATLAB software package and subjected to wavelet analyzer analysis.

For the selection of the mother wavelet, we visually examined the obtained signal for a best match with the available standard mother wavelets. By this visual inspection, it is found that the ‘‘db4″ mother wavelet is potentially most similar to the measured AE signatures shown in Figs. 5, 6 and 7. This observation is highly consistent with the outcomes of references^44^^,^^47^^,^^48^, and^49^.

**Figure 9:**
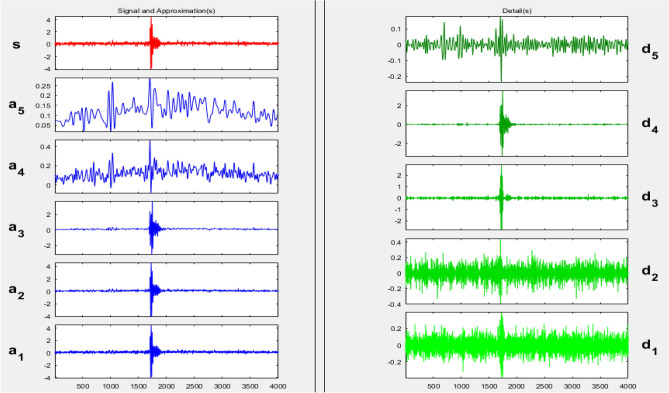
DWT separate mode decomposition for one PD pulse of broken disc.

**Figure 10:**
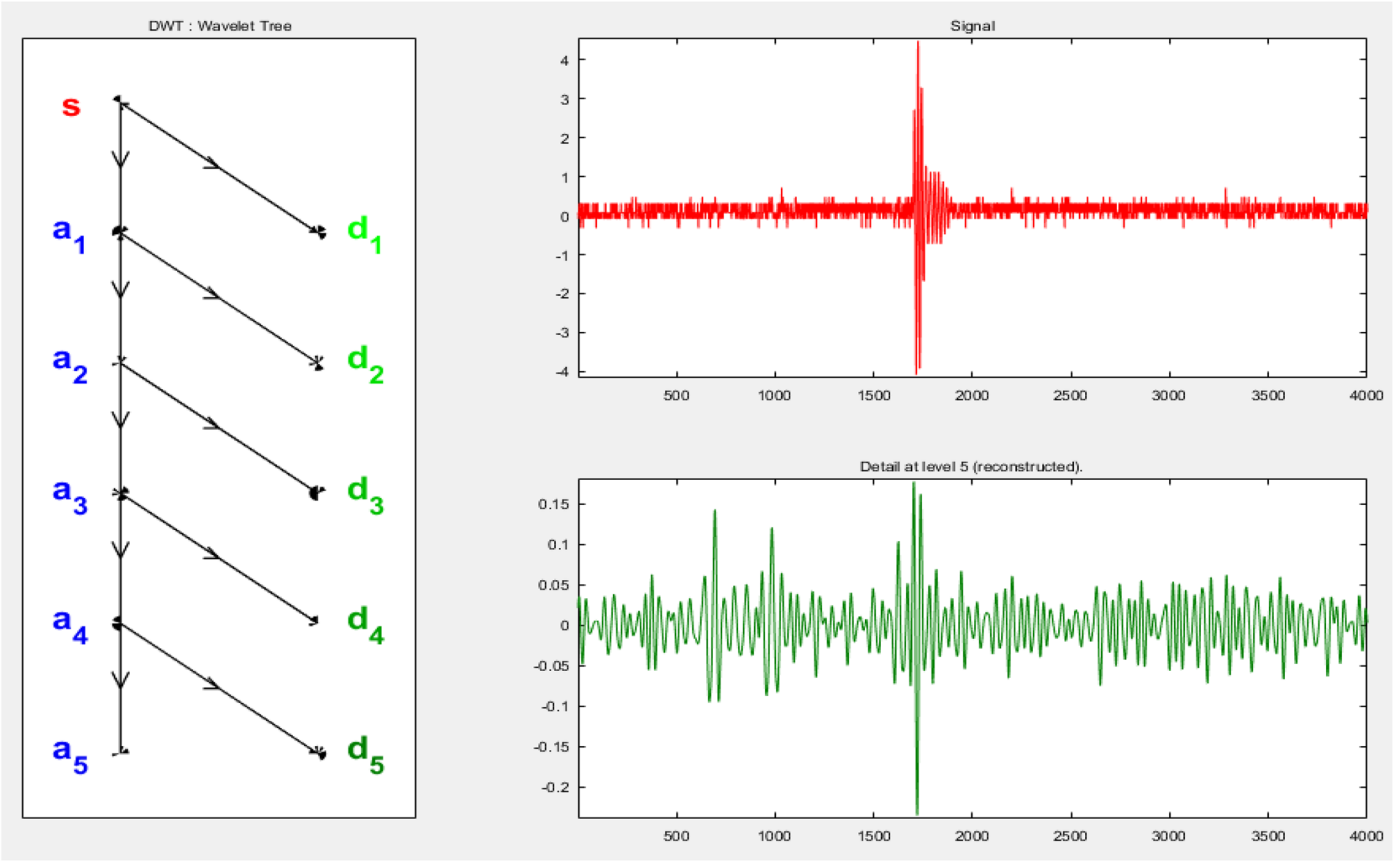
Tree mode decomposition for one PD pulse of broken disc.

Each captured signal is decomposed into 5-levels of approximations, which contain low frequency components, and other 5-levels of details, which contain high frequency components using the “db4” as a mother wavelet. This process is very difficult to be done manually for each signal of 100 PD pulses and repeated for all cases. So, a MATLAB code for DWT was designed to be used at any time for all sample cases. Figures 9 and 10 show the wavelet transform decomposition, in a MATLAB environment, of one PD acoustic wave captured with a broken insulator in separate and tree decomposition modes, respectively. Finally, each PD test signal in the wavelet analyzer is decomposed into five distinct decomposition signals using the below equation:

6 $${S={a}_{5}+d}_{1}+ {d}_{2}+ {d}_{3}+ {d}_{4}+ {d}_{5}$$

where: S is the main signal, a_5_ is the 5^th^ level in approximation components, and d_1_, d_2_, d_3_, d_4_, d_5_ are the 5-levels in details respectively.

The seven features under investigation were cleverly extracted from the decomposed approximation and detail signals using the DWT m-file algorithm. For example, Table 1 provides the features that were extracted from the 6-decomposed components (a_5_, d_1_, d_2_, d_3_, d_4_, d_5_) of Fig. 6 in the time domain. These decomposed signals related to a single PD pulse collected from the broken insulator. This study focuses on the relative change in the estimated feature values as an indicator of the type of defect present rather than on the absolute values of these features. Likewise, an algorithm has been devised to extract and compute the features of the decomposed signals for the 100 PD signal waves acquired from each of the three samples studied in this work.

**Table 1:** Extracted features for a single wave of the broken sample.

Features	Levels					
	a_5_	d_1_	d_2_	d_3_	d_4_	d_5_
M	6.63E-04	1.59E-05	3.09E-05	−7.03E-08	−1.76E-05	1.19E-05
V	5.82E-08	1.34E-08	2.15E-08	2.97E-07	9.32E-07	3.37E-08
Max	0.0015	5.50E-04	5.76E-04	0.0055	0.0064	9.60E-04
Min	1.80E-04	−5.07E-04	−6.77E-04	−0.0059	−0.0108	−6.09E-04
m_e_	4.97E-07	1.37E-08	2.25E-08	2.96E-07	9.28E-07	3.36E-08
S	0.7710	−0.1474	0.0248	0.5437	−4.866	0.5395
K	3.691	3.531	3.767	78.16	74.07	7.874

ANOVA analysis test is included in the m-file MATLAB code for minimizing the extracted features/levels and selecting the significant features/levels from the seven original features. Table 2 shows the estimated *P*-value for each feature/level. Each calculated *P*-value in that table shall be compared to 0.05, and the decision rule here is that if the computed *P*-values are less than 0.05, the corresponding features/levels are considered significant and could be ignored otherwise. The lowest two values are chosen for each corresponding feature. Consequently, two levels are selected from each extracted feature to be the most significant. Finally, 14-levels/features, as shown in Table 3, are selected and ready to be used as input data to the neural network for identification of the type of defects in the insulators.

**Table 2:** *P*-values of ANOVA test for each feature/level at 95% confidence.

Features	Levels					
	a_5_	d_1_	d_2_	d_3_	d_4_	d_5_
m	**6.88E-52**	0.410	0.278	0.518	0.020	**1.41E-06**
V	0.217	**9.77E-47**	0.059	0.161	0.0004	**4.10E-11**
Max	**4.39E-25**	0.022	1.28E-10	0.0003	2.79E-09	**1.25E-12**
Min	**2.01E-21**	0.280	8.82E-10	0.013	0.025	**1.78E-12**
m_e_	**5.89E-45**	**4.55E-45**	0.001	0.160	0.0004	1.13E-10
S	0.686	0.084	**0.005**	0.152	0.060	**3.35E-07**
K	0.204	**3.74E-13**	1.59E-11	**5.61E-21**	0.003	2.28E-10

**Table 3:** Selected features to be inputs to the classifier.

Features	Levels	
m	a_5_	d_5_
V	d_1_	d_5_
Max	a_5_	d_5_
Min	a_5_	d_5_
m_e_	a_5_	d_1_
S	d_2_	d_5_
K	d_1_	d_3_

The outcomes of the ANOVA test are highly consistent with the findings obtained through the F-value, J-value and B-value criteria, which were discussed in^1,51^, and^52^, respectively.

### Artificial neural network

**Figure 11:**
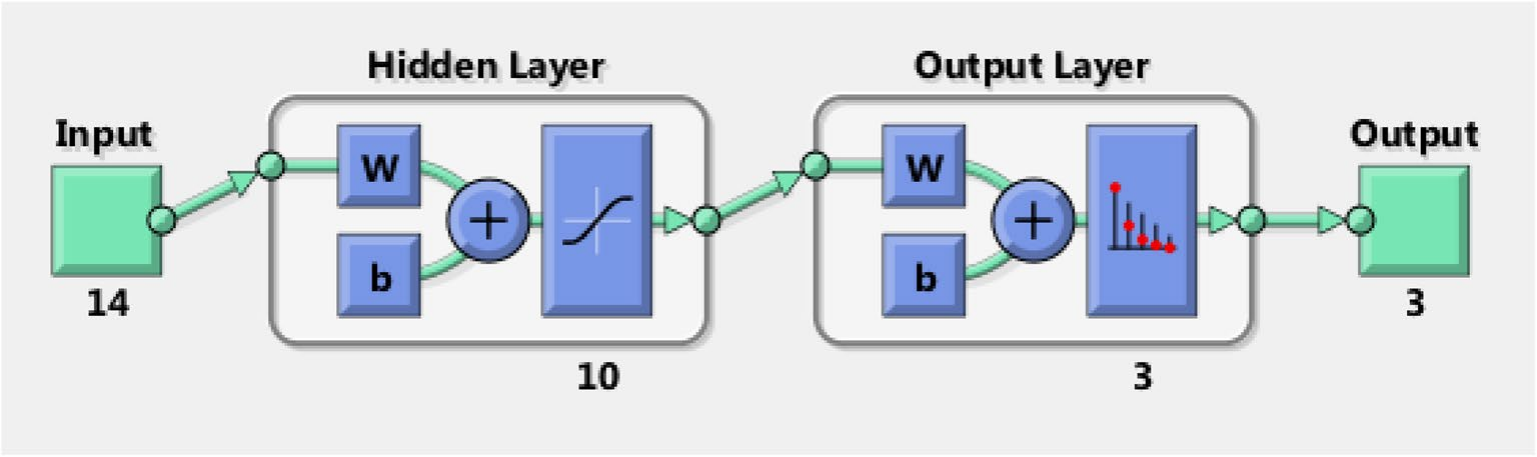
ANN architecture for DWT.

According to feature selection analysis, two levels were selected for each feature of the extracted seven features to be input data to the classifier, which is known as ANN as shown in Table 3. So, the input data is a matrix of 14 × 300 representing 300 samples of 14 elements, and the target (output) data is proposed to be 3 × 300 matrix representing 300 samples of 3 elements, as shown in Fig. 11. In this classifier, the 300 samples are randomly divided into 70% (210 samples) for training, 15% (45 samples) for the validation and 15% (45 samples) for testing. It has been observed from the results that the best performance of validation process is achieved at error 0.0816. This error is calculated as the deviation between the target (the proposed output of the ANN) and the actual output of the ANN itself. Further, the obtained overall recognition rate of these data is 68.119%. This obtained result is considered poor in the classification process indicating low accuracy of the designed classifier. In order to overcome this problem, authors have decided to increase the dataset for the ANN by collecting more acoustic emission signals to improve the performance of the classifier. So, the experimental process was repeated to obtain 200 acoustic signals for each test sample and data processing techniques were performed on 200 signals for each sample instead of 100 signals.

**Figure 12:**
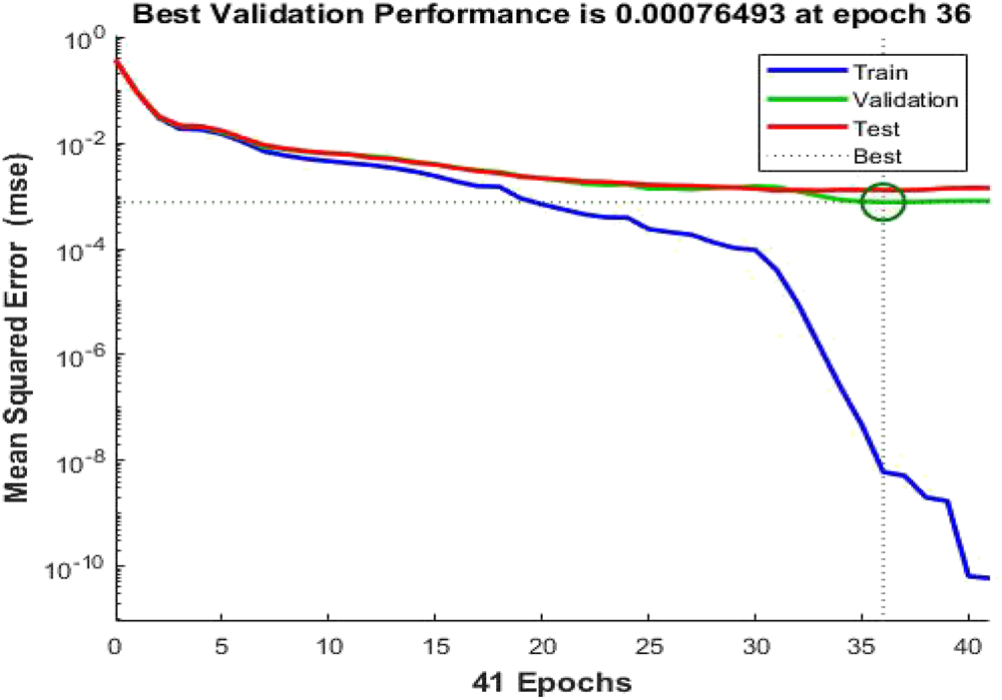
Performance plot of ANN for DWT.

**Figure 13:**
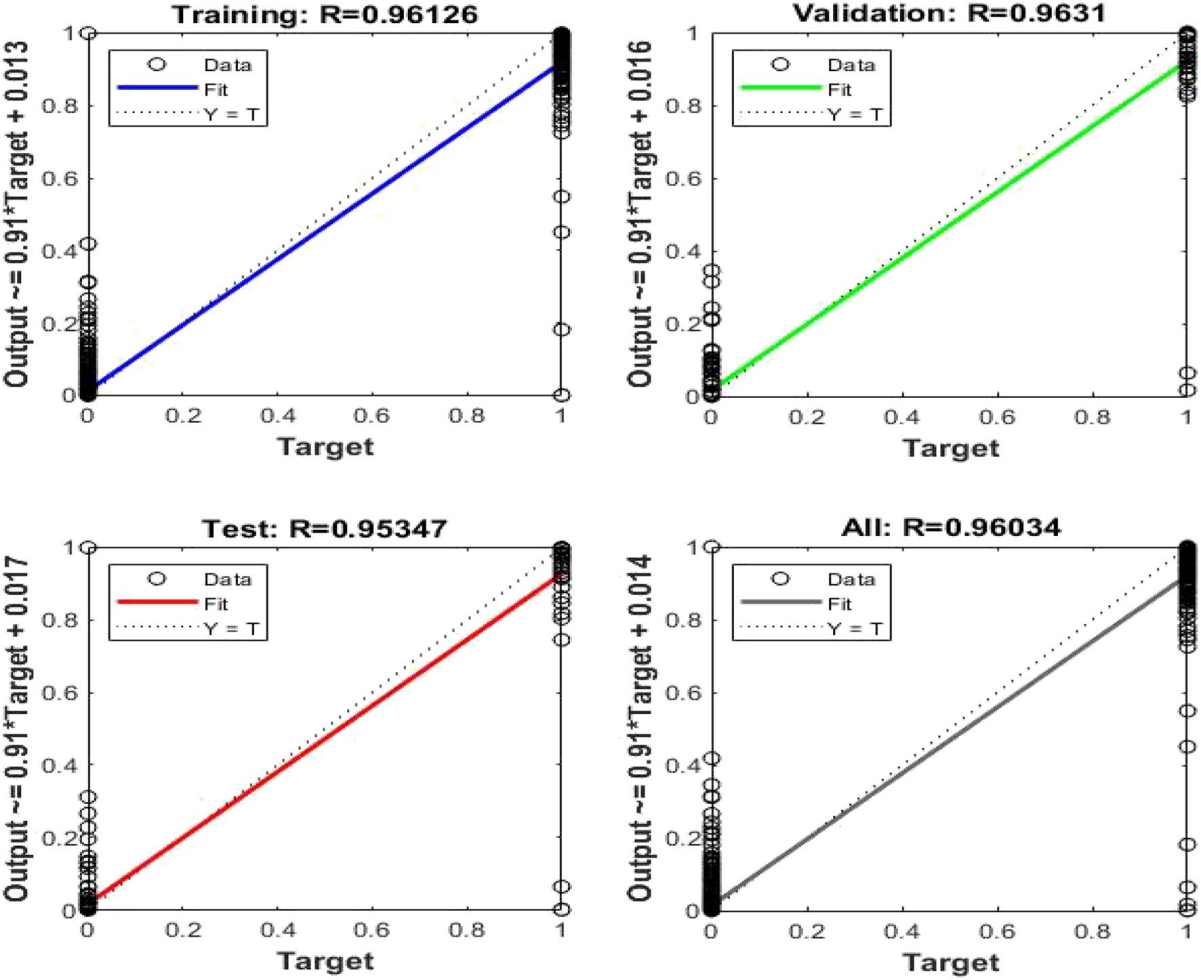
Regression plot of ANN for DWT.

Now, the input data becomes a matrix of 14 × 600 and the target data is proposed to be a matrix of 3 × 600. Figure 12 shows that the best validation performance is obtained at 0.000765 mean squared error (MSE). This small error indicates the high performance of the developed classifier. Figure 13 shows a regression plot of training, validation, testing and the overall recognition rate of the data. It has been seen that the overall recognition rate of the classifier is 96.034% indicating a high accuracy and performance of the classifier.

**Figure 14:**
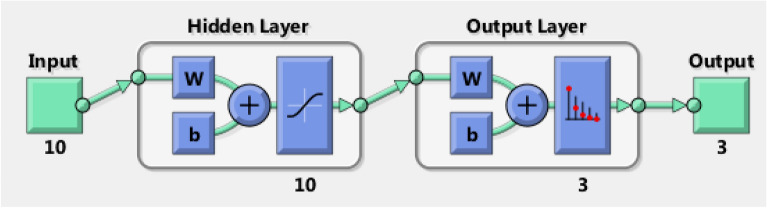
ANN architecture for FFT.

**Figure 15:**
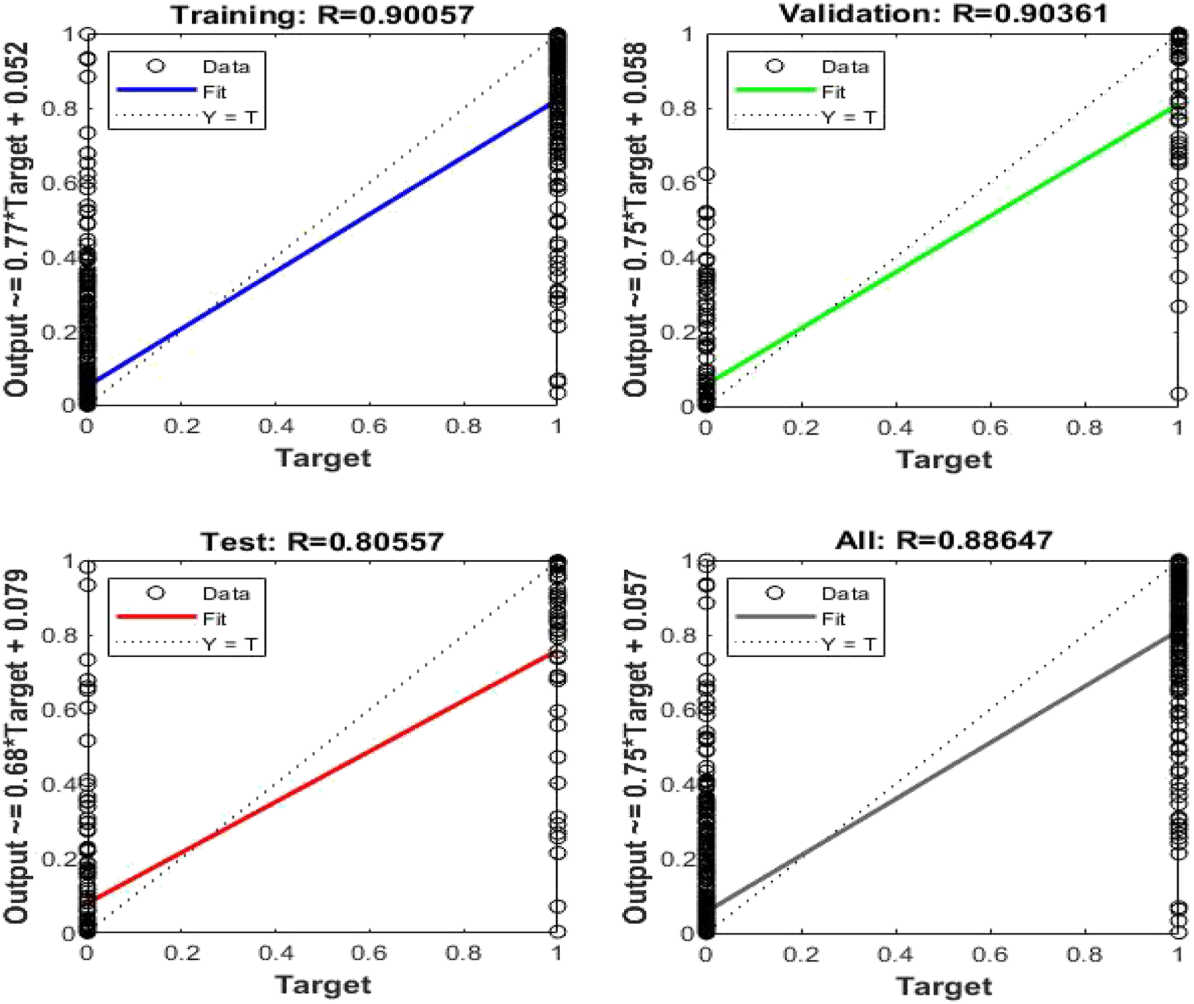
Regression plot of ANN for FFT.

On the other hand, from FFT analysis, the first ten peak values of each pulse are assumed to be input data to the ANN. So, the input data is a matrix of 10 × 600 and the output data is proposed to be the same as the previous classifier’s 3 × 600 matrix, as shown in Fig. 14. With this tool, the best validation performance is achieved at error 0.031838. In addition to that, the overall accuracy recognition rate is obtained at 88.647% as illustrated in Fig. 15.

In conclusion, it has been observed, as cleared in Table 4, that the recognition rate obtained from DWT is higher than that obtained from FFT analysis. Additionally, the best validation performance is achieved with a lower error in the wavelet transform than in the FFT. Consequently, DWT analysis is more accurate and is recommended for use in classifying defects in ceramic outdoor insulators.

**Table 4:** Comparison between DWT and FFT.

Technique	Validation performance error	Recognition rate
DWT	0.000765	0.96034
FFT	0.031838	0.88647

## Validation of results

It is important to validate the findings of the Artificial Neural Network (ANN) to provide an alternative perspective on the classification performance and help assess the robustness of the ANN results. In this context, support vector machine (SVM) and K-nearest neighbors (KNN) are both employed to validate the findings obtained from ANN. SVM is a supervised learning algorithm that aims to find an optimal hyperplane to classify data points into different classes. KNN, on the other hand, is a non-parametric algorithm that classifies data points based on their proximity to the nearest neighbors in the training set.

**Figure 16:**
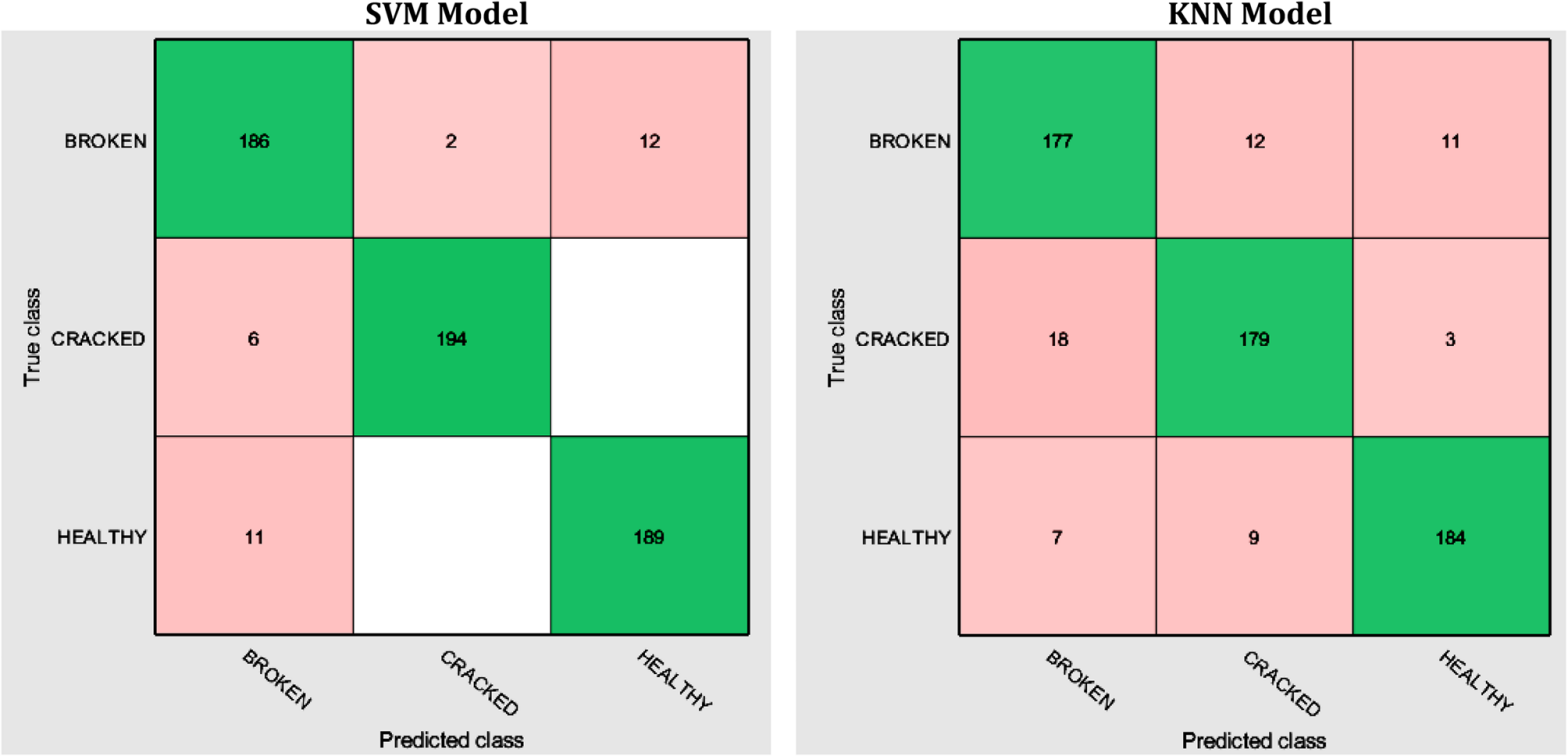
Confusion matrix of SVM and KNN models.

SVM and KNN models are implemented using classification learner application in a MATLAB environment. The input dataset, obtained from the feature selection stage, was prepared and imported for training. Based on the results, the average value of SVM accuracy is 94.8% and KNN accuracy is 90.0%. These results can be observed from the confusion matrix for each model, as shown in Fig. 16. It has been observed that the achieved classification accuracy is high, reaching or exceeding 90.0%. Furthermore, it has been noted that the classification accuracies of these models are relatively close to each other, indicating a degree of consistency in their predictions. Therefore, the outcomes of the used validation algorithms increase confidence in the accuracy, stability, and reliability of the results.

## Research contribution

- The current research discusses an online safety monitoring technique for ceramic outdoor insulators with actual defects that could happen during service with high-bandwidth equipment.
- In data processing techniques, the ANOVA test was introduced as an advanced tool for feature selection process, and its findings were matched with those of other tools used before.
- It has been observed from ANN results that the overall recognition rate depends on the number of collected signals. It implies that a greater number of signals captured results in a higher recognition rate.
- SVM and KNN algorithms were used to validate the outcomes of the proposed ANN technique. It has been found from the results that the ANN, SVM, and KNN models have matched with each other and are suitable tools for the classification of defects in high voltage outdoor ceramic insulators.

## Conclusion

Detection and monitoring of PD is a significant diagnosis of defects in outdoor ceramic insulators for ensuring reliability and preventing any interruption in the electrical power network. As a result, variable defects in ceramic insulators have been studied in this current paper, which are known to be sources of PD activities. A DAS has been established for capturing and recording the acoustic emission signals resulting from PD activities in defective ceramic insulators. DWT has been introduced for extracting features from the captured signals and decomposing the original signals into multi-level signals to get more information about the acoustic signatures for each test sample. The ANOVA test has been adopted and used as a feature selection tool. Two levels for each extracted feature have been selected to be the most significant signatures for the classification of defective insulators and are ready to be the input dataset to the neural network, which is known as a classifier. ANN is used in this current work for classifying defects in ceramic insulators. It has been observed from ANN results that the overall recognition rate depends on the number of collected signals. It implies that a greater number of signals captured results in a higher recognition rate. The overall recognition rate is obtained at 96.03% from DWT and 88.65% from FFT, indicating a high accuracy and performance classifier. SVM and KNN models are used to validate the findings of the ANN technique. It has been observed that the classification accuracy of these models is relatively close to each other, indicating a degree of consistency in their predictions. Therefore, the outcomes of the used validation algorithms increase confidence in the accuracy, stability, and reliability of the results. It is concluded that a successful neural network analysis for the classification of defected ceramic insulators could have important practical applications for the safety and reliability of the electrical power transmission and distribution system.

## Data Availability

The data sets used and/or analyzed during the current study are available from the corresponding author on reasonable request.
